# Chondral and Soft Tissue Injuries Associated to Acute Patellar Dislocation: A Systematic Review

**DOI:** 10.3390/life11121360

**Published:** 2021-12-08

**Authors:** Filippo Migliorini, Emanuela Marsilio, Francesco Cuozzo, Francesco Oliva, Jörg Eschweiler, Frank Hildebrand, Nicola Maffulli

**Affiliations:** 1 Department of Orthopedic, Trauma, and Reconstructive Surgery, RWTH Aachen University Hospital, 52064 Aachen, Germany; joeschweiler@ukaachen.de (J.E.); fhildebrand@ukaachen.de (F.H.); 2 Department of Orthopaedics, Surgery and Dentistry, University of Salerno, Via S. Allende, 84081 Baronissi, Italy; emarsilio@unisa.it (E.M.); fcuozzo@unisa.it (F.C.); foliva@unisa.it (F.O.); n.maffulli@qmul.ac.uk (N.M.); 3 Centre for Sports and Exercise Medicine, Barts and the London School of Medicine and Dentistry, Mile End Hospital, Queen Mary University of London, 275 Bancroft Road, London E1 4DG, UK; 4 School of Pharmacy and Bioengineering, Keele University Faculty of Medicine, Thornburrow Drive, Stoke on Trent ST5 5BG, UK

**Keywords:** first time patellar dislocation, chondral lesion, MPFL rupture, soft tissue

## Abstract

Introduction: Chondral and soft tissue injuries can be associated with first time patellar dislocation, but it is unclear how common they are, and which tissues are affected. A systematic review of the literature was performed to investigate the frequency, location, and extent of chondral and medial patellofemoral ligament (MPFL) injuries in patients following first time patellar dislocation. Methods: This systematic review was conducted according to the PRISMA guidelines. PubMed, Google Scholar, Embase, and Web of Science databases were accessed in November 2021. All the published clinical studies reporting the frequency, location, and extent of soft tissue lesions following first time patellar dislocation were accessed. Studies reporting data on habitual, congenital, or recurrent patellofemoral instability were excluded. Results: Data from 42 articles (2254 patients, mean age 21.6 ± 7.3 years) were retrieved. Ninety-eight percent of patients who experienced first time patellar dislocation demonstrated MPFL rupture at MRI. Forty-eight percent of MPFL ruptures were located at the patellar side, 34% at the femoral insertion site, and 18% in the midportion. Eighty-five percent of patients showed signs of patellar chondral damage at MRI, and trochlear chondral injuries were evidenced in 47% of patients. Intra-articular loose bodies were observed in 11.5% of patients. At arthroscopy, the medial facet and the crest of the patella more commonly exhibited chondral lesions than the lateral facet and femoral trochlea. Conclusions: Most patients suffer chondral damage and MPFL tears following after a first time patellar dislocation.

## 1. Introduction

Acute patellar dislocation is common, especially in adolescents [1]. Acute patellar dislocations account for approximately 3% of all knee injuries [2,3]. Up to 45% of patients who experience an acute patellar dislocation develop recurrent patellofemoral instability [4,5]. Patellar tracking is ensured by dynamic and static restrains to lateral displacement of the patella [6]. Most patients who experience a patellar dislocation present two or more concomitant pathoanatomical risk factors which synergistically interact and predispose to patellofemoral instability [7,8,9,10]. Trochlear and patellar dysplasia, patella alta, mal alignment syndromes (e.g., varus/valgus deformity, greater tibial extrarotation, and femoral head anteversion), hyperlaxity, female gender, and family history of patellofemoral instability are the major predisposing factors to patellofemoral instability [11,12,13].

The frequency, location, and extent of chondral injuries, along with the rate of medio patellofemoral ligament (MPFL) tears following first time patellar dislocation are unclear [14,15,16,17,18]. Soft tissue injuries can occur during reduction of the patellar dislocation [19,20,21]. Pathoanatomical factors predisposing to patellofemoral instability can be demonstrated in most patients with patellar chondral defects [22,23]. If left untreated, chondral defects lead to persistent pain, limiting daily living activities, and may induce premature osteoarthritis in the long term [24,25,26,27]. The MPFL is the most important restraint to the lateral displacement of the patella during the first 30 degrees of flexion; thus, if left untreated, MPFL tears can increase joint instability and consequently the risk of redislocation [28,29,30,31]. Better characterization of soft tissue injury patterns in patients following first time patellar dislocation may assist in establishing proper treatment. A systematic review of the literature was performed to investigate the frequency, location, and extent of chondral injuries and MPFL tears in patients after first time patellar dislocation. We hypothesized that most patients following first time patellar dislocation demonstrate chondral and MPFL damages.

## 2. Material and Methods

### 2.1. Search Strategy

This systematic review was conducted according to the Preferred Reporting Items for Systematic Reviews and Meta-Analyses (PRISMA) guidelines [32]. The literature search was guided by the following points:

- Problem: first time patellar dislocation;
- Outcome: soft tissue injuries.

### 2.2. Literature Search

Two authors (**;**) independently performed the literature search in November 2021. PubMed and Google Scholar were accessed. Embase and Web of Science were also accessed to identify further articles. The following keywords were used in combination: *patella, dislocation, acute, instability, soft tissue, chondral, cartilage, lesion, osteochondral, injury, loose body, ligament, tear, muscle injury, tendon tear, ACL, PCL, MCL, meniscus, MRI, arthroscopy, medial patellofemoral ligament, MPFL, damage, insertion, rupture, Outerbridge, International Cartilage Repair Society, ICRS.* The same authors performed the screening of the resulting titles in a separate fashion, accessing the full-text of the articles of interest. A cross-reference of the bibliographies was also performed. Disagreements were resolved by a third author (**).

### 2.3. Eligibility Criteria

All the published clinical studies which reported the frequency, location, and extent of chondral injuries and MPFL ruptures following first time patellar dislocations were accessed. Given the authors language capabilities, articles in English, German, Italian, French, and Spanish were eligible. Level I to IV of evidence, according to Oxford Centre of Evidence-Based Medicine [33], were considered. Reviews, technical notes, comments, letters, editorials, protocols, and guidelines were not eligible, nor were biomechanical, animal, and cadaveric studies. Studies reporting data on habitual, congenital, or recurrent patellofemoral instability were excluded. Studies involving patients who underwent previous patellofemoral surgical procedures were also not eligible. Only articles reporting quantitative data under the outcomes of interest were considered for inclusion.

### 2.4. Outcomes of Interest

Data extraction was performed by two authors (**;**). Study generalities were collected: author, year, journal, study design, number of patients. MRI findings were retrieved: rate of free loose bodies, rate, extent, and location of MPFL, trochlear, and patellar chondral injuries. Arthroscopy findings were also collected: location and extent of trochlear and patellar chondral injuries. The MRI findings were evaluated using the Outerbridge classification [34], whereas the International Cartilage Repair Society (ICRS) [35] was used for the arthroscopic findings.

### 2.5. Methodology Quality Assessment

Two authors (**;**) independently performed the methodological quality assessment using the PEDro Score. PEDro is an 11-item scale designed for rating methodological quality of the included studies. These items evaluated the eligibility criteria, allocation and blinding procedures, the length of the follow-up, the intention-to-treat and between-group analyses, point estimates, and variability. Items were scored either as present (1) or absent (0). Mean values of 6/11 were considered satisfactory.

### 2.6. Statistical Analysis

For the statistical analysis, IBM SPSS (Chicago, IL, USA) software version 25 was used. For continuous variables, the Shapiro–Wilk test was performed to investigate data distribution. For normal data, mean and standard deviation were calculated. For nonparametric data, median and interquartile range were calculated. Dichotomic data was evaluated as follow:

(1) $$\frac{Number\;of\;patients\;reporting\;the\;event}{Number\;of\;patients\;included\;in\;the\;study}\times 100$$

## 3. Results

### 3.1. Search Result

**Figure 1:**
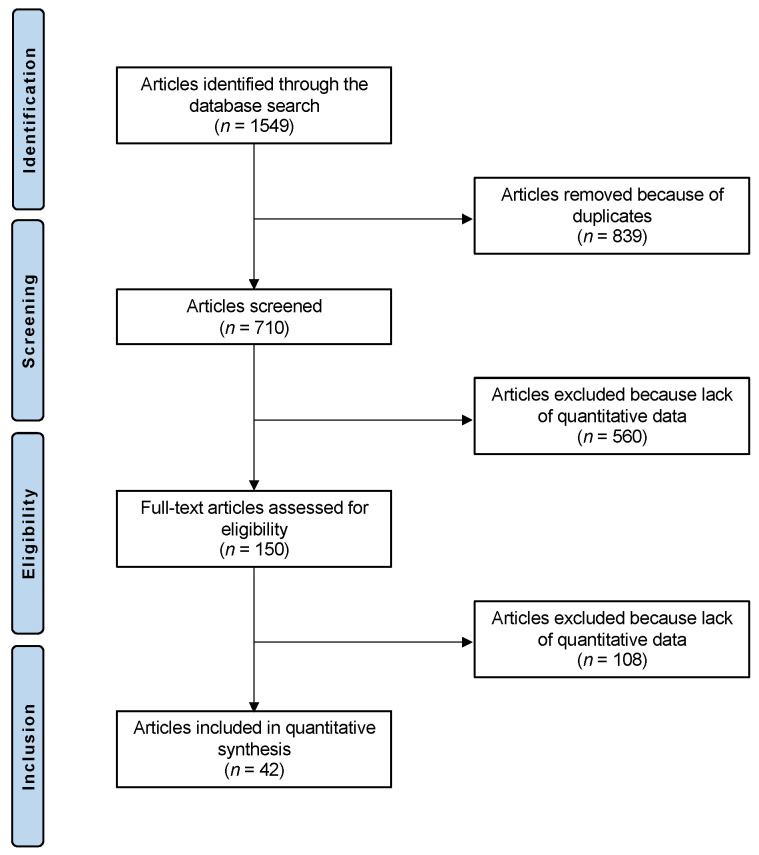
Flowchart of the literature search.

The literature search resulted in 1549 articles. Of these, 839 were excluded as duplicates. Another 560 were not eligible due to not matching the topic (*n* = 250), study design (*n* = 201), recurrent, congenital, or habitual patellofemoral instability (*n* = 103), language limitation (*n* = 4), and uncertain results (*n* = 2). This left 150 articles for inclusion. A further 108 articles were excluded because of lack of quantitative data under the outcomes of interest. Finally, 42 articles were considered for analysis (Figure 1).

### 3.2. Methodological Quality Assessment

The adequate baseline comparability, length of the follow-up, and outcome measure reliability were the most important strengths of this study. The most important limitations evidenced by the PEDro score were the lack of randomization and blinding, along with the high risk of bias during allocation concealment. In conclusion, the PEDro score resulted in 6.6/11, attesting to the acceptable quality of the methodological assessment (Table 1).

### 3.3. Patient Demographics

A total of 2254 patients were retrieved. The mean age was 21.6 ± 7.3 years old. The generalities of the included studies are shown in Table 2.

### 3.4. MRI Findings

Twenty-two studies (1002 patients) [14,38,40,41,43,46,47,48,53,56,57,58,60,63,64,65,67,68,70,71,72,73,74,75] investigated the status of MPFL at MRI. Ninety-eight percent (1031 of 1052) of patients demonstrated MPFL rupture following first time patellar dislocation. The patellar side was damaged in 48.0% (315 of 656), 33.7% (268 of 402) the femoral site, and 18.2% (73 of 402) the midportion. Eighteen studies (1487 patients) [14,37,40,41,45,52,53,54,55,56,58,61,62,66,67,71,72,73] reported the presence of loose bodies. Overall, 11.5% (183 of 1589 patients) showed loose bodies. Four studies (410 patients) [64,72,74,75] evaluated 191 chondral injuries (47%) of the lateral trochlea using the Outerbridge classification. These injuries were classified as grade I (6.2%), II (17.5%), III (9.3%), IV (10.3%), and V (11.3%). Three studies (100 patients) [56,69,72] evaluated 85 chondral injuries (85%) of the patella using the Outerbridge classification. These injuries were classified as grade I (17.6%), II (32.4%), III (20.3%), IV (31.1%), and V (0%). These results are shown in greater detail in Table 3.

### 3.5. Arthroscopic Findings

Ten studies (236 patients) [15,37,50,51,52,55,60,63,66,67,68] evaluated the chondral lesions arthroscopically using the IRCS (Table 3). In the lateral patellar facet, 2.8% of lesions were grade I lesions, 2.8% grade II, 2.8% grade III, but none were grade IV. In the medial patellar facet, 2.8% of lesions were grade I, 18.3% grade II, 15.5% grade III, and 29.6% were grade IV. In the median crest, 7.0% of lesions were grade I, 19.7% grade II, 8.5% grade III, and 18.3% grade IV. In the trochlea, 9.7% of lesions were grade I, 13.9% were grade II, 3.2% were grade III, but none were grade IV. These results are shown in greater detail in Table 4.

According to the main findings of the present systematic review, 98% of patients who experienced first time patellar dislocation demonstrated MPFL rupture at MRI. This rupture is more frequent at the patellar site (48%), whereas femoral (34%) and midportion (18.2%) tears are less common. Eighty-five percent of patients following patellar dislocation reported signs of patellar chondral damage at MRI, whereas 47% demonstrated trochlear chondral injuries. Arthroscopic findings evidenced that the medial chondral facet and median crest of the patella were affected more commonly by advanced chondral injuries than the lateral patellar facet and femoral trochlea. Loose bodies were observed in 11.5% of patients.

The patellofemoral joint is complex, with intricate architecture and biomechanics. Following patellar dislocation, ruptures of the MPFL have been demonstrated in 81% to 100% of patients [76,77,78,79,80,81,82]. Tears at the patellar insertion site were more common (47% to 76%), whereas femoral (26% to 49%) and midportion (13% to 30%) tears are less frequent [14,40,83]. The current evidence concerning the location of the MPFL tear are controversial. Several authors found a predominance of ruptures located at the femoral site [14,84,85]. Sallay et al. [60] evidenced that the MPFL was ruptured at the femoral side in 87% of patients, whereas in only 4% of patients, the tears were located at their patellar insertion [60]. Similarly, Sillanpaa et al. [86] found that 57% of patients have tears of MPFL at the femoral site, 23% mid-substance, and 20% at the patellar site.

The exact incidence of chondral defects after patellar dislocation is unclear. Up to 96% of patients demonstrated cartilage injuries after the first episode of patellar dislocation [14,15,16,17]. Guerrero et al. [14] evaluated injuries after traumatic lateral patellofemoral dislocation on 195 patients. They found a rate of chondral damage of 49% (96 of 105 knees) at MRI [14]. Nomura et al. [87] arthroscopically evaluated the cartilage status of 70 patients after patellar dislocation. They found that 96% (67 of 70 knees) had patellar cartilage injuries: fissures were observed in 75% (53 of 70 knees), fibrillation or erosion in 77% (54 of 70 knees) [87]. Sanders et al. [16] found that 40% (10 of 25 knees) demonstrated trochlear chondral damages: 70% (7 of 10 patients) full-thickness chondral defects with subchondral bone exposure, 30% (3 of 10 patients) with subchondral bone damage [16]. Whether these patients require a combined chondral procedure (e.g., AMIC, MFx) has still not been clarified.

Given the multifactorial aetiology along with the juvenile onset, the management of patellar dislocation can be challenging [88,89]. Primary patellar dislocations are typically managed conservatively, and surgery is reserved for patients with recurrent patellar dislocations or demonstrating loose bodies or osteochondral defects [13,26,90]. The rate of redislocation after conservative management ranges from 15% to 71% [28,29,62,91,92,93,94,95,96,97,98,99]. Conservative treatments included braces, cryotherapy, physiotherapy, and long periods of absence from sports [26,27]. Previous clinical studies comparing surgical versus conservative management for first patellofemoral dislocations suggest that patients may benefit from prompt surgery immediately after the first acute patellar dislocation [86,100,101,102,103,104,105,106,107,108]. In a recent meta-analysis including 654 patients, immediate surgery was compared to conservative management for acute patellar dislocation [31]. At a mean of 54 months follow-up, the risk of redislocation was 2.44 folds greater in the conservative group, along with a 10% worse Kujala score [31]. If left untreated, patellofemoral instability can result in anterior knee pain, persistent instability sensation, and remarkable reduction in quality of life [109,110,111]. Therefore, a growing tendency to surgically treat the first patellar dislocation has been evidenced [112,113]. Moreover, isolated reconstruction of the MPFL shows excellent results and patient satisfaction, together with a very low rate of complications and failure [114]. Indeed, centres performing MPFL reconstruction have doubled in the last decades [115]. Concluding, patients may benefit from prompt surgical intervention to repair soft tissue injuries and restore patellar tracking, preventing further redislocations. An MRI should be considered in all the patients following patellar dislocation to evaluate soft tissue injuries.

The present investigation has certain limitations. The retrospective design and the relatively short length of follow-up in most available studies represents important limitations. Most of the included studies were case series or case reports, which lead to an increased risk of selection and allocation biases, negatively impacting the conclusions of the present systematic review. A formal control group was missing in most studies. Elias et al. [40] included a control group of consecutive knee MR imaging examinations performed for various indications. A control group of patients suffering recurrent patellar dislocations were used in two studies [55,69]. Sanders et al. [61] matched their patients with a cohort of patients who did not experience a previous patellar dislocation. No study took advantages from blinding methods, increasing the risk of detection and performance biases. The primary aim of most studies was not to report the rate of soft tissue injuries, which could represent an important source of bias. Most of the authors referred to “MPFL rupture” without clarifying the extent (partial or total tears). Given these limitations, results from the present study must be interpreted with caution. Future studies should validate these results in larger scale investigations.

## 4. Conclusions

Almost all patients following first time patellar dislocation presented chondral damages and MPFL rupture.

## Figures and Tables

**Table 1:** Methodological quality assessment.

PEDro Score	
1. Eligibility criteria	98% (41 of 42)
2. Random allocation	0% (0 of 42)
3. Concealed allocation	43% (18 of 42)
4. Baseline comparability	86% (36 of 42)
5. Blind subject	43% (18 of 42)
6. Blind clinician	0% (0 of 42)
7. Blind assessor	21% (9 of 42)
8. Adequate follow-up	93% (39 of 42)
9. Intention-to-treat analysis	95% (40 of 42)
10. Between-group analysis	90% (38 of 42)
11. Point estimates and variability	74% (31 of 42)

**Table 2:** Generalities and patient demographics of the included studies.

Author, Year	Journal Name	Design	Knees (*n*)	Mean Age (Mean)
Bui et al., 2008 [36]	*Skeletal Radiol*	RCS	20	31
Callewier et al., 2009 [37]	*Orthop Traumatol Surg Res*	CR	1	23
Cavalheiro et al., 2018 [38]	*Rev Bras Ortop*	CR	1	13
Cho et al., 2009 [39]	*J Manipulative Physiol Ter*	CR	1	16
Elias et al., 2002 [40]	*Radiology*	RCS	82	20
Felus et al., 2008 [41]	*J Pediatr Orthop*	PCS	22	14
Gesslein et al., 2019 [42]	*Cartilage*	RCS	53	19.5
Gormeli et al., 2016 [43]	*J Pak Med Assoc*	CR	1	41
Guerrero et al., 2009 [14]	*Sports Med Arthrosc Rehabil Ther Technol*	RCS	195	23
Jabalameli et al., 2017 [44]	*J Surg Case Rep*	CR	3	17
Jalan et al., 2014 [45]	*J Clin Diagn Res*	CR	1	30
Kang et al., 2018 [46]	*Chin Med J (Engl)*	RCS	43	14.1
Lee et al., 2013 [47]	*Knee Surg Sports Traumatol Arthrosc*	RCS	9	14.6
Lee et al., 2020 [48]	*J Orthop Surg (Hong Kong)*	CR	1	16
Maleki et al., 2019 [49]	*Biomed Res Int*	CS	17	14.1
Maletius et al., 1994 [50]	*Arthroscopy*	CR	2	19.5
Mashoof et al., 2005 [51]	*Arthroscopy*	RCS	7	24
Megremis et al., 2019 [52]	*J Am Acad Orthop Surg Glob Res Rev*	CR	1	14
Nakagawa et al., 2014 [53]	*Case Rep Orthop*	CR	2	14.5
Nietosvaara et al., 1994 [54]	*J Pediatr Orthop*	PCS	72	13.3
Nomura et al., 2003 [15]	*Arthroscopy*	CS	39	18
Nomura et al., 2005 [55]	*Osteoarthritis Cartilage*	RCS	30	17.2
Paakkala et al., 2010 [56]	*Skeletal Radiol*	CS	23	19–45
Park et al., 2020 [57]	*JBJS Case Connect*	CR	1	15
Pedowitz et al., 2019 [58]	*Am J Sports Med*	RCS	41	13.8
Quinn et al., 1993 [59]	*J Magn Reson Imaging*	CS	8	
Sallay et al., 1996 [60]	*Am J Sports Med*	RCS	12	25
Sanders et al., 2017 [61]	*Am J Sports Med*	PCS	609	21.4
Sanders et al., 2018 [62]	*Knee Surg Sports Traumatol Arthrosc*	PCS	232	14.1
Saragaglia et al., 2020 [63]	*Int Orthop*	PCS	39	23
Seeley et al., 2012 [64]	*J Pediatr Orthop*	RCS	111	14.9
Seeley et al., 2013 [65]	*J Pediatr Orthop*	RCS	46	14.6
Stanitski 1995 [66]	*Am J Sports Med*	CS	17	13.8
Stanitski et al., 1998 [67]	*Am J Sports Med*	CS	48	14
von Engelhardt et al., 2010 [68]	*BMC Muskuloskelet Disord*	CS	40	21.5
Vollnberg et al., 2012 [69]	*Eur Radiol*	RCS	51	22.4
Wissmann et al., 2009 [70]	*J Comput Assist Tomogr*	RCS	14	33
Wilson et al., 2013 [71]	*Orthop J Sports Med*	CS	36	14.5
Zaidi et al., 2006 [72]	*Pediatr Radiol*	RCS	26	13.9
Zhang et al., 2013 [73]	*Injury*	RCS	49	24.5
Zhang et al., 2015 [74]	*Injury*	PCS	121	25
Zheng et al., 2015 [75]	*Injury*	PCS	127	14.1

**Table 3:** MRI findings.

First Time Patellar Dislocation—MRI Findings	
Evidence of lateral trochlea damage (Outerbridge)	47% (191 of 410)
	I (6.2%)
	II (17.5%)
	III (9.3%)
	IV (10.3%)
	V (11.3%)
Evidence of patellar damage (Outerbridge)	85% (85 of 100)
	I (17.6%)
	II (32.4%)
	III (20.3%)
	IV (31.1%)
	V (0%)
Free loose bodies	11.5% (183 of 1589)
MPFL tear	98% (1031 of 1052)
patellar site	48.0% (315 of 656)
femoral site	33.7% (268 of 402)
midportion	18.2% (73 of 402)

**Table 4:** Arthroscopic findings (ICRS: International Cartilage Repair Society) discussion.

First Time Patellar Dislocation—Arthroscopic Findings	
Lateral patellar facet (ICRS)	I (2.8%)
	II (2.8%)
	III (2.8%)
	IV (0%)
Medial patellar facet (ICRS)	I (2.8%)
	II (18.3%)
	III (15.5%)
	IV (29.6%)
Median crest lesions (ICRS)	I (7.0%)
	II (19.7%)
	III (8.5%)
	IV (18.3%)
Trochlea (ICRS)	I (9.7%)
	II (13.9%)
	III (3.2%)
	IV (0%)

## Data Availability

The data presented in this study are available within the article.
